# Novel online Recommendation algorithm for Massive Open Online Courses (NoR-MOOCs)

**DOI:** 10.1371/journal.pone.0245485

**Published:** 2021-01-22

**Authors:** Asra Khalid, Karsten Lundqvist, Anne Yates, Mustansar Ali Ghzanfar

**Affiliations:** 1 School of Engineering and Computer Science, Victoria University of Wellington, Wellington, New Zealand; 2 School of Education, Victoria University of Wellington, Wellington, New Zealand; 3 Department of Computer Science, The School of Architecture, Computing and Engineering, University of East London, London, United Kingdom; Hefei University of Technology, CHINA

## Abstract

Massive Open Online Courses (MOOCs) have gained in popularity over the last few years. The space of online learning resources has been increasing exponentially and has created a problem of information overload. To overcome this problem, recommender systems that can recommend learning resources to users according to their interests have been proposed. MOOCs contain a huge amount of data with the quantity of data increasing as new learners register. Traditional recommendation techniques suffer from scalability, sparsity and cold start problems resulting in poor quality recommendations. Furthermore, they cannot accommodate the incremental update of the model with the arrival of new data making them unsuitable for MOOCs dynamic environment. From this line of research, we propose a novel online recommender system, namely NoR-MOOCs, that is accurate, scales well with the data and moreover overcomes previously recorded problems with recommender systems. Through extensive experiments conducted over the COCO data-set, we have shown empirically that NoR-MOOCs significantly outperforms traditional KMeans and Collaborative Filtering algorithms in terms of predictive and classification accuracy metrics.

## 1 Introduction

Since the advent of the first MOOCs in 2008 [1], MOOCs have added a new dimension to education systems. The main attraction for learners is the access to free open education courses. The number of MOOCs and the number of students registered in MOOCs are growing every year using platforms such as edX, Coursera, Udacity, NetEase and iCourse [2]. By the end of 2019, more than 900 universities were offering MOOCs with 13500 courses available, and around 110 million registered students [3], providing learners with a wide variety of choices. With such a high number of courses available, learners face the problem of selecting courses without being overwhelmed by multiple learning choices [4], which has been termed as the information overload [5] problem. Information filtering systems also known as recommender systems can overcome this problem by helping learners to find suitable courses from the space of available resources.

With the increased usage of MOOCs, data produced by MOOCs is also increasing. This data contain information about the interests and behaviors of learners and courses in which they are registered [6]. Recommender systems have been used widely by commercial and social platforms [4] and recommender systems can use educational data to provide recommendations to learners [7]. One purpose of these recommender systems is to help the learner by recommending different related learning objects or elements. As well as improving the learners’ experience, recommender systems have also played a vital role in increasing the popularity of MOOCs and much of the previous research work has focused on the design of such recommender systems. Research to date has mostly focused on the implementation of recommender systems in MOOCs, particularly course recommender systems which has been the most prolific research line since 2012 [8].

Recommender systems consist of two basic elements: items and users. Users give their rating about items, and the recommender system gives recommendations to users for new items [9]. We denote users by $U=\{u_{1},u_{2},\cdots ,u_{M}\}$, here *M* is the total number of users using the system i.e. |U|=M. The Term $I=\{i_{1},i_{2},\cdots ,i_{N}\}$ is the set of all the items where |I|=N where *N* is total number of items in the system. By using users and items, the system maintains profiles. The user profile is defined by its characteristics such as ratings provided on items, age, gender, and location. Each user has a unique identification to discriminate them from other users [9]. Items are defined by their characteristics as with the course recommender system the characteristics of each course are name, description, category and so on.

Each user gives ratings to some of the items in the system. These ratings are represented by $\left(r_{iu}|\left(i,u\right)\in D\right)$; here D⊂I×U is the set of user-item pair that have been rated. |D|=T denotes the total number of ratings made by users. Generally, each user rates a small number of items, so |D|=T≪|I×U|=N. In a real data-set, it is common to have *T*/(*M* × *N*) ≅ 0.01. A (*M* × *N*) matrix *R* is called a user-item rating matrix. Ratings given by users can be considered as elements of this matrix *R*.

Although a considerable number of recommender systems in MOOCs have been proposed and implemented, only a few authors have discussed the time and space complexity of their proposed and implemented algorithms [4, 10–13]. MOOCs produce a large amount of data that can be used for recommender systems and researchers should focus on developing systems that scale well with the increase in data. The aim of this study is to contribute to the development of an accurate and practical algorithm for MOOCs which will utilize rating data of learners for recommendations. The proposed algorithm is a fast rating based recommender algorithm that can work with large data sets. The proposed algorithm processes each data point only once, so it requires less memory and time. It is a local learning algorithm [14], which is similar to Platt [15] and a modified model of Bassam [16]. It is efficient, scalable, incremental and generalizes well for large data sets.

The rest of the paper is structured as follows. Section 2 provides an overview of the literature pertaining to recommender systems in MOOCs. Section 3 describes the recommendation algorithm. Section 4 discusses the experimental setup and results of algorithm on the COCO data-set. Section 5 concludes the research findings and discusses future work.

## 2 Related work

As discussed above, the implementation of course recommender systems is a key focus of the existing research on recommender systems in MOOCs [8]. A possible reason for this focus could be the availability of data and the interests of MOOC providers because course recommender systems can help improve both the enrolment numbers and students’ learning experiences. Most of the work on the implementation of recommender systems for courses uses traditional recommendation methods which include content-based filtering (CBF) and collaborative filtering (CF) [17]. Previous studies also have introduced association rule mining and hybrid algorithms [18, 19] along with collaborative and content-based filtering for course recommendation [11, 20].

Boratto et. al [20] used collaborative filtering, matrix factorization and hybrid recommendation for calculating recommendations to learners. The purpose of this work was to identify the effect of an item’s popularity on recommendation results and the research showed popular items effect the recommendation list of the recommender system.

Yanhui et. al [21] used content based and collaborative filtering recommender systems to firstly categorise courses on the basis of course information, then courses and learners are divided into fuzzy clusters with collaborative filtering being used for clustering. Each course and learner can be linked in more than one cluster, and if a learner and a course are in more than one similar cluster it means that they are similar and that the learner could be interested in that course. A score is maintained for each course on the basis of similarity between the course and the learner. Then a score based top down ranking of courses is listed.

The algorithm designed in [22] considered user profile and performance while making recommendations. Instead of considering all users, this algorithm considered only similar users while performing a recommendation. This recommender system was designed separately from the website leaving the burden of recommendation on the system. This system consists of four parts. In the first part, the system performs implicit rating based on user learning behavior and stores them. Then by using these ratings, the system finds a neighboring cluster using learner data. Then the system is trained to find feature vectors and the parameter vector, which are then used for recommendations. This system uses implicit rating which is not reliable because with a change in the model of rating generator, ratings will change as well.

Ahera et al. [10] used K-means for clustering and an Apriori rule algorithm to recommend courses to students. In this work, only those courses were considered which had more than 100 students registered and only those students who had registered in at least five courses. There was no consideration given to new users and item scenarios. The recommendation system in [23] utilized association rules mining to analyse learner activities. The Parallel FP-growth algorithm of MLlib machine learning library was used to design recommendation system. A decentralized approach was used for data processing and analysis to get a better performance.

Symeonidis et al. [24] designed a course recommender system using a multi-dimensional matrix, such as matrix factorization with collaborative filtering called xSVD++. This algorithm uses information from external sources, user skills and course characteristics to predict course trends and ratings. This algorithm uses course ratings, learner skills and course skills to perform recommendations.

Apaza et al. [25] matched the syllabus of colleges with other courses available on MOOCs. Latent Dirichlet Allocation (LDA) is used to infer topics from the college syllabus and MOOCs. These topics and grading information are used by a content based recommendation system to calculate recommendations. This algorithm can only provide recommendations to students who have grading and college syllabus information.

More recently, researchers have started to use neural networks, pattern mining, and deep learning for pre-processing of data and recommendation [8]. Campos et al. implemented a course recommender system using knowledge re-use in ecosystems and [26] considered the context of the learner while performing recommendations. Obeidat et al. [27] utilized student course history to find similar students and designed a collaborative recommender system that uses data-mining techniques for pattern identification between course. Clustering of students is performed on the basis of their history which is then used to generate association rules through an Apriori algorithm. This work showed that the usage of user study history for rule generation produces good results, but this system cannot be used for new users without any history.

Zhang et al. [28] used deep belief networks (DBN) to propose a personalized course recommendation system. In his work he used DBN for recommendation generation, feature extraction and classification. Through DBN, users’ course interest data is mined and utilized for recommendation.

Sabnis et. al [17] carried out an analysis of the importance of course recommender systems by analyzing different recommendation techniques that are used for course recommendation. According to this study CF and CBF cannot give results in the case of new users. They concluded that every approach has pros and cons so selecting an approach totally depends upon the scenario for which it will be used. They suggested hybrid approaches can be used to get the benefits of different approaches.

Researchers have also designed recommender systems for different types of learning elements, such as video clips, next page and additional helpful resources to the learner. Kopeinik [29] compared existing recommender algorithms that provide a recommendation of learning resources and tags to annotate these resources. Onah and Sinclair [30] recommended a suitable learning path to learners by considering scores on concept-based quizzes; a low score indicated that the learner needed to read more resources related to a concept, so this system recommended instructional material to learners according to their profile.

Although a considerable number of recommender systems in MOOCs have been proposed and implemented, only a few have discussed the time and space complexity of their proposed and implemented algorithms [4, 10, 11, 13]. MOOCs produce a large amount of data that can be used for recommender systems and researchers should focus on systems that scale well with the increase in data. One reason for overlooking this aspect is that research has mostly focused on offline processing of data. Batch/offline algorithms use existing data-sets for training and recommendations. Time and memory space required for the training process is dependant upon the type of data-set that is being used.

Only a few researchers have used ratings for recommendation of courses and learning elements. Ratings can help recommending courses when other data about learners and courses are not available. The present research work aims to design a scalable and practical generalized rating based recommender system that will use ratings given by learners or by the MOOC provider to any element (e.g. course, learning element, video, peer) in the MOOC to generate recommendations.

## 3 Novel online Recommendation algorithm for Massive Open Online Courses (NoR-MOOCs)

This section explains the proposed algorithm for MOOCs named as the ‘Novel Online Recommendation Algorithm for Massive Open Online Courses (NoR-MOOCs)’. It is a fast rating based recommender algorithm that can work with large data sets. NoR-MOOCs processes each data point only once hence it requires less memory and time. It is a local learning algorithm which is similar to Platt [15] and a modified model of Bassam [16] and [31]. It is efficient, scalable, incremental and generalizes well while handling large data sets.

Before going into the details of the proposed algorithm, some important concepts used in this algorithm are explained. They are voting, hyper-spheres and generations. This algorithm groups data points in the form of hyper-spheres where every hyper-sphere has a dynamic radius, which is computed after each generation. These hyper-spheres are fuzzy and can overlap.

### 3.1 Voting

Voting is used for compression of the data-set. In voting, an active learner is compared with the center of every hyper-sphere. Voting gives results in the form of weights -1 to 1; where -1 shows that learners and a hyper-sphere are opposite to each other and 1 shows both are similar [32]. During the voting process, a weight is assigned to every hyper-sphere on the basis of the value of the distance between an active learner and a hyper-sphere. That weight value is then used to compute the final recommendation for the learner.

### 3.2 Hyper-sphere

Each hyper-sphere represents a group of similar learners. Each hyper-sphere has a center, density of hyper-sphere and a dynamic radius which is re-calculated after every generation.

- **Center**: Every hyper-sphere has a center which contains a record of the number of times every course is rated by all learners and the average rating given by each learner to the courses. It is updated when a new learner is added into the hyper-sphere.
- **Density of hyper-sphere**: Density of hyper-sphere is the number of learners in every hyper-sphere. It shows the size of the hyper-sphere.
- **Radius**: This algorithm maintains a dynamic radius of every hyper-sphere. Radius is used to define a boundary around the hyper-sphere. The value of the radius is used to decide whether or not a point will become part of the hyper-sphere or not. When a new point arrives, the distance of that point from the center is measured and compared with the value of radius of that hyper-sphere. If the radius is larger than the distance, then that point falls into the currently considered hyper-sphere.
- **Generation**: Generation is a defined size of points. After processing a certain number of points, the algorithm re-defines the radius of the hyper-spheres. This makes it possible that every hyper-sphere has a different radius. Generation is a parameter that is tuned for the algorithm.

### 3.3 Training process

For the first learner (data point), there will be no hyper-sphere available, so the algorithm computes the center of hyper-sphere from the first learner and set its radius to a default value (see section 4.2.1). For subsequent learners, there will be two scenarios, either the learner resides in one or many existing hyper-spheres or they reside outside of all existing hyper-spheres.

- If the learner resides in existing hyper-spheres and has similarity with one or more, then that learner is added into all of the similar hyper-spheres and the center, radius and density of all the hyper-spheres are updated accordingly.
- If the learner resides outside of the existing hyper-spheres, then a new hyper-sphere is created by setting that point as center of that hyper-sphere. The average of the radii of all existing hyper-spheres becomes the radius of the new hyper-sphere.

### 3.4 Recommendation process

While computing recommendations, a learner can reside in one, many or no hyper-spheres. In the case of a learner being similar to one or many hyper-spheres, then the most similar hyper-sphere is selected. In this situation, the value of the distance between the learner and the hyper-spheres is normalised by dividing it with the density of selected hyper-spheres. Recommendations are then computed for the learner by using the weighted average of all the similar hyper-spheres. In the situation where there is no similar (distance of learner from center of hyper-sphere is more than the radius) hyper-sphere, the most similar hyper-sphere will be selected from all existing hyper-spheres and the value of the distance between the learner and the hyper-spheres will be normalised by dividing it by the density of that hyper-sphere. Recommendations are then computed by using the weighted average of all the similar hyper-spheres.

### 3.5 Example case study

To understand the working of the proposed algorithm, a step by step process is demonstrated below through an example.

#### 3.5.1 Training process

**Fig 1:**
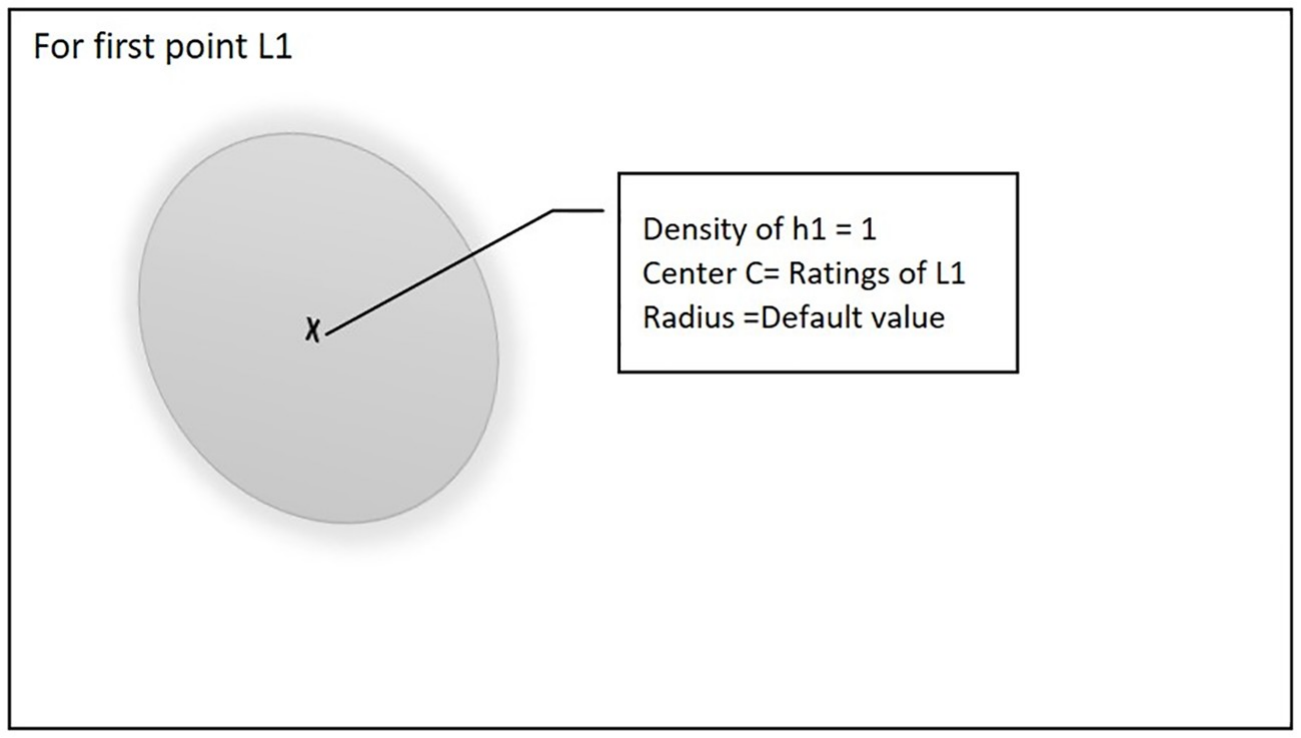
The first point will become the center of the hyper-sphere.

Fig 1. shows that at the start of training process when there is no existing hyper-sphere NoR-MOOCs will create a new hyper-sphere and set the first point L1 as the center of the hyper-sphere.

**Fig 2:**
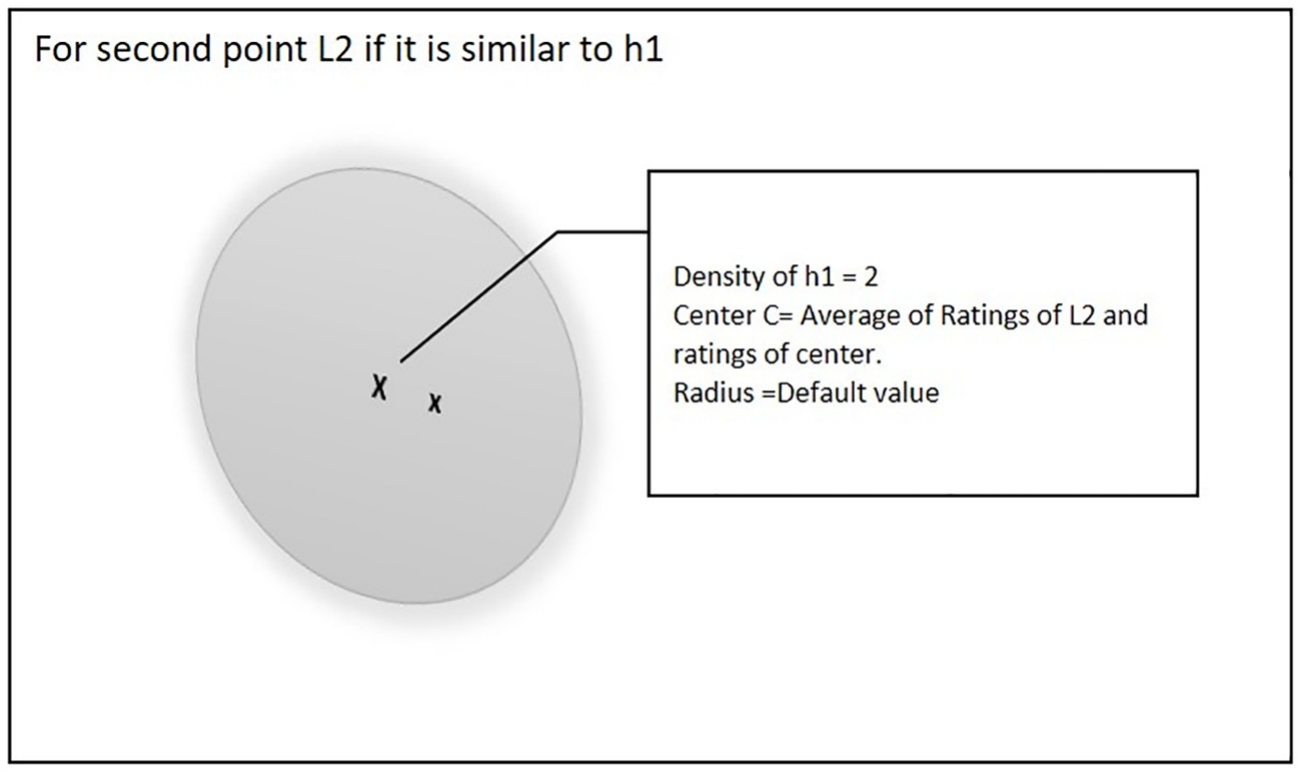
Second point is not similar to existing hyper-sphere.

When the second point L2 arrives during the training process, there will be two possible scenarios. Fig 2 shows the first scenario, if point L2 is not similar to an existing hyper-sphere then a new hyper sphere will be created and L2 will be set as the center of that hyper-sphere.

**Fig 3:**
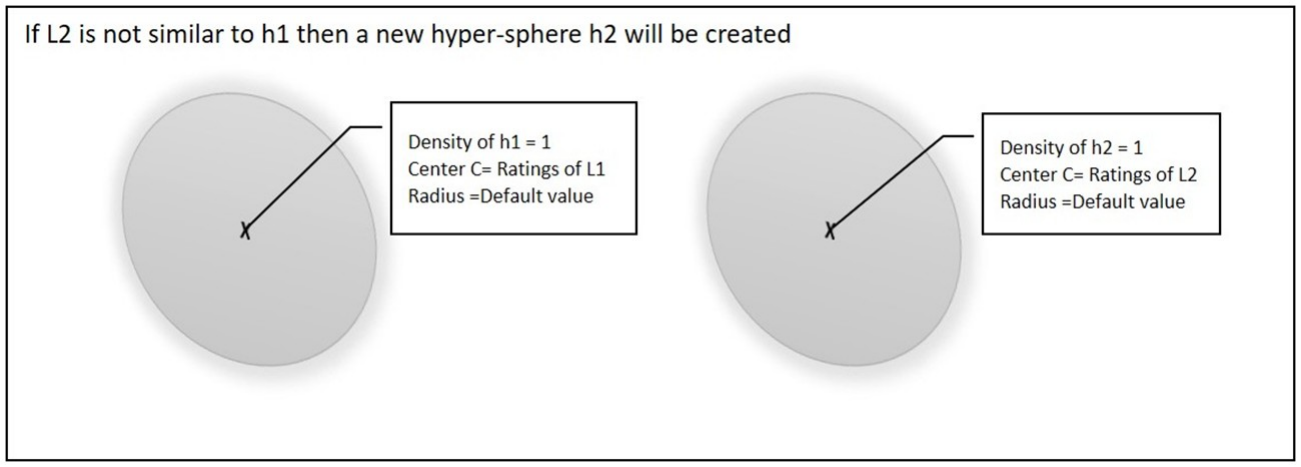
Second point similar to existing hyper-sphere.

Fig 3 shows the second scenario, if point L2 is similar to an existing hyper-sphere, then the center of that hyper-sphere will be updated and the density of hyper-sphere will be incremented by 1.

**Fig 4:**
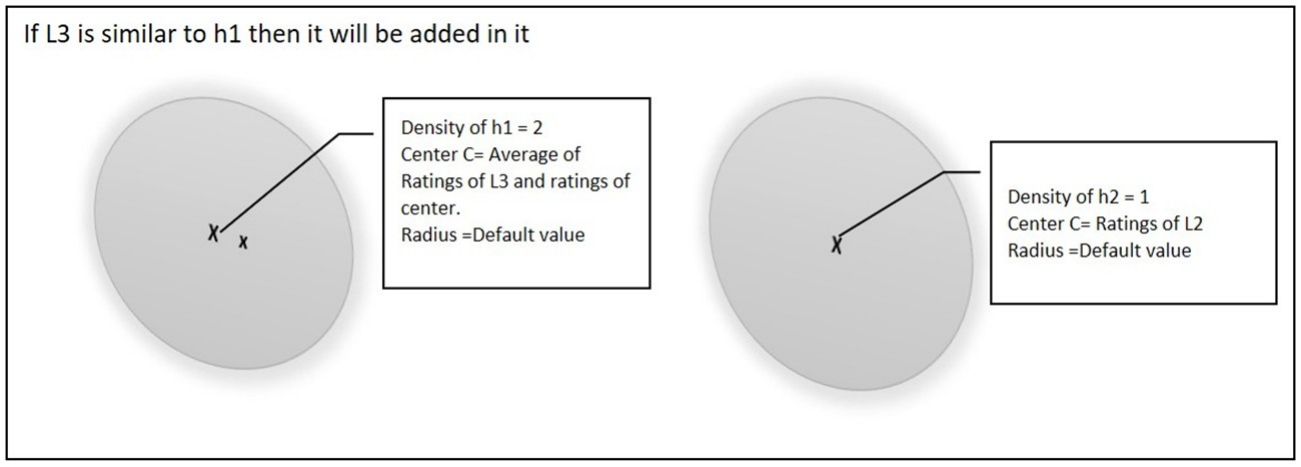
Third point is similar to one of the existing hyper-spheres.

When the third point L3 arrives then there will be three possibilities. Fig 4 shows the first case if point L3 is similar to one of the existing hyper-spheres (e.g. h1). Then the center of that hyper-sphere will be updated and the density of the hyper-sphere will be incremented by 1.

**Fig 5:**
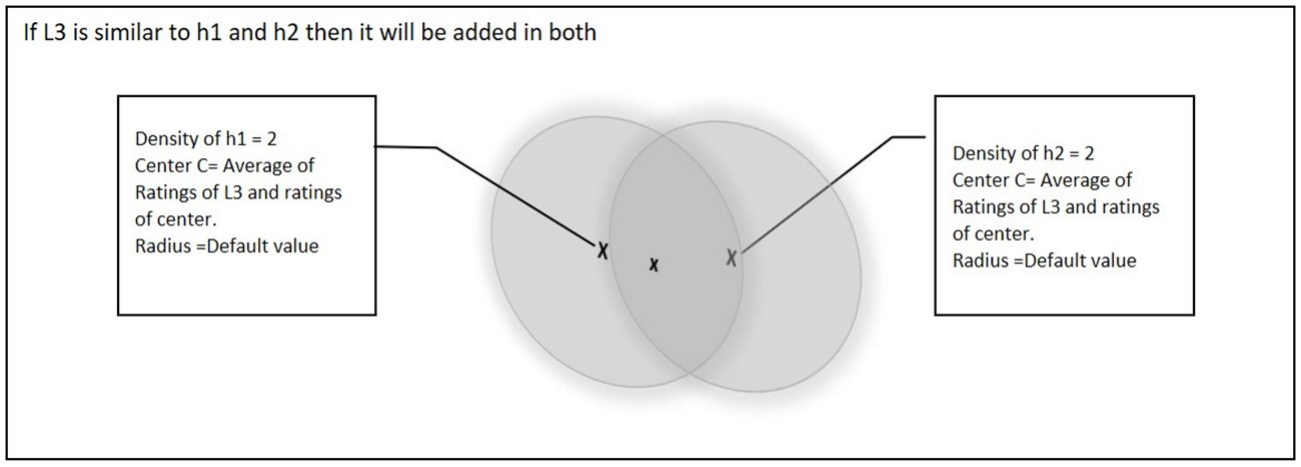
Third point is similar to both of the existing hyper-spheres.

Fig 5 shows a further scenario if point L3 is similar to both of the existing hyper-spheres (i.e. h1 and h2). In this situation, the center of both hyper-spheres will be updated and the density of the hyper-spheres will also be incremented by 1.

**Fig 6:**
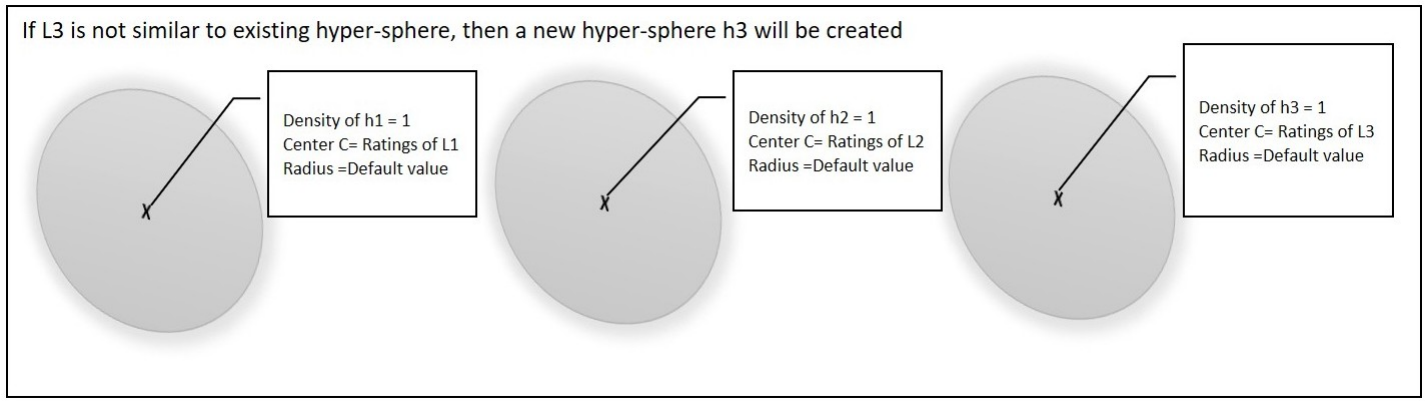
Third point is not similar to any of the existing hyper-spheres.

Fig 6 shows the third case, if point L3 is not similar to any of the existing hyper-spheres then a new hyper-sphere will be created and L3 will be set as the center of that hyper-sphere.

**Fig 7:**
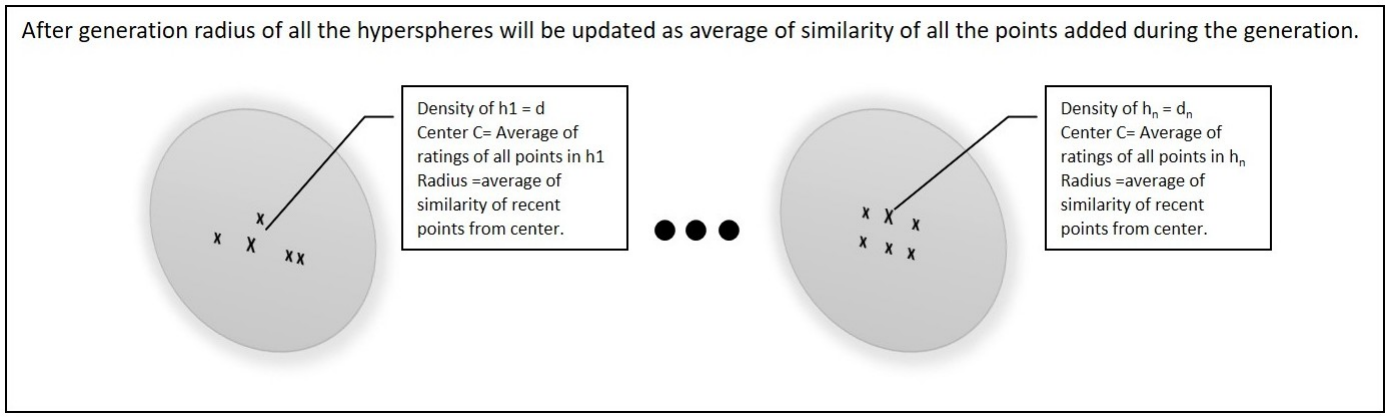
Radius of all the existing hyper-spheres will be updated after generation.

For all the remaining points in the training data-set the same scenario will work as of third point L3 until the number of processed data-points equal to generation size. After that the radius of every existing hyper-sphere will be updated as the average of similarity of all the points that were added into the hyper-sphere during that generation as shown in Fig 7.

#### 3.5.2 Recommendation process

**Fig 8:**
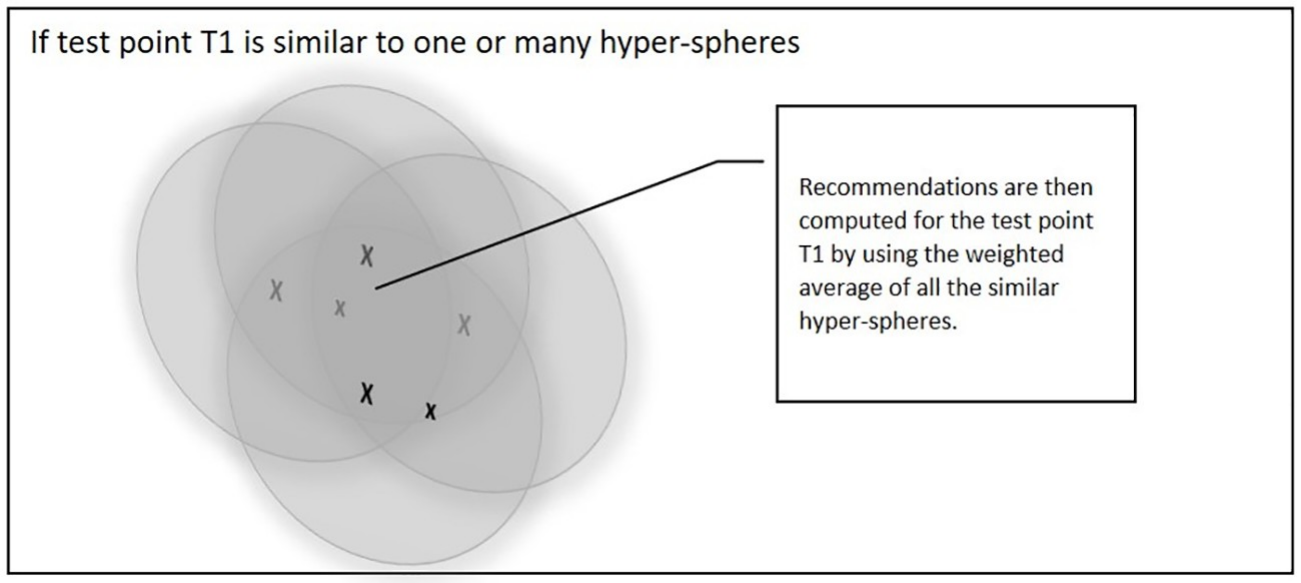
Test point T1 is similar to one or many hyper-spheres.

During the recommendation process for testing point T1, there are two possibilities. Fig 8 shows the first case if test point T1 is similar to one or many hyper-spheres then the value of the distance between T1 and the hyper-spheres is normalised by dividing it by the density of the selected hyper-spheres. Recommendations are then computed for T1 by using the weighted average of all the similar hyper-spheres.

**Fig 9:**
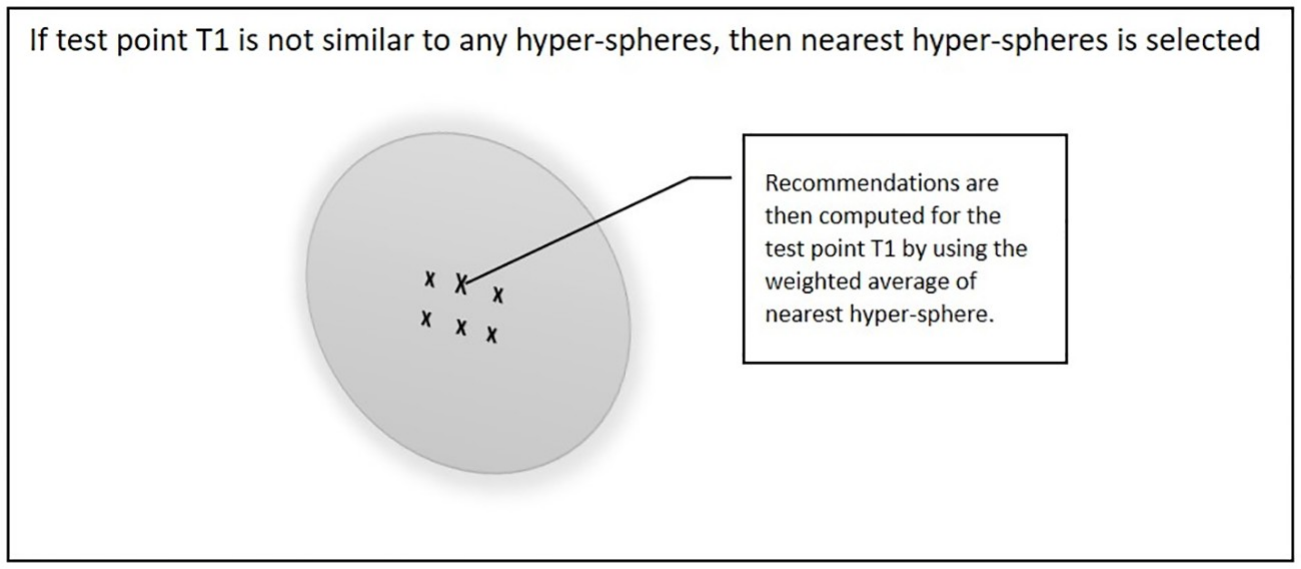
Test point T1 is not similar to any of hyper-spheres in the training set.

Fig 9 shows the second case if test point T1 is not similar to any of the hyper-spheres in the training set, then the nearest hyper-sphere will be selected. In this situation, the value of the distance between the T1 and hyper-spheres is normalised by dividing it by the density of the selected hyper-spheres. Recommendations are then computed for the T1 by using the weighted average of the hyper-spheres.

### 3.6 NoR-MOOCs adaption with an increase in the data-set

An important feature of NoR-MOOCs is that it can work well with new data and doesn’t need any re-training. In the case of a new data point, it compares it with existing hyper-spheres and if the new data point is similar to any of the existing hyper-spheres, it adds the new data point to that hyper-sphere; otherwise it creates a new hyper-sphere. In this way it updates the existing trained model without the need to re-train the whole model.

Algorithms 1 and 2 outline the training and recommendation processes respectively.

Let *l* ∈ *D* represents all learners and *h* ∈ *H* represents all hyper-spheres. In the beginning, the hyper-sphere is empty i.e. *H* = *ϕ*, so the first point will make first the hyper-sphere. The Center *c* of the first hyper-sphere will be created using the first point. It is assumed that each hyper-sphere shows a specific group *g* (*g* ∈ *G* where *G* represents the set of all groups) of learners having similar interests.

### 3.7 Distance between learner and hyper-sphere

In Eq 2, the term *d*(*c*_*h*_, *l*_*n*_) denotes the distance between learner *l*_*n*_ and the center of hyper-sphere *c*_*h*_. For computing distance, we compute similarity between *c* and *l*_*n*_. These two values are inverse of each other, hence if the similarity of two points has a high value it means that distance between them will be zero. The similarity function is shown as follows:

$$\begin{array}{c} \text{Sim}=\{\begin{array}{cc} \frac{1}{\text{Dis}} & if\;\text{Dis}\neq 0\;,\\ {\text{Max}}_{\text{Sim}} & otherwise, \end{array} \end{array}$$ (1)

Here *Max*_*Dis*_ shows the maximum distance or zero similarity between any two points. In Pearson’s Correlation, results can be negative, hence 1 was added to all the similarities to make the results positive. For more information, please refer to our previous work [33].

**Algorithm 1 NoR-MOOCs training**

**Input**: labeled training set < (*l*_1_, *g*_1_), (*l*_2_, *g*_2_), … (*l_n_*, *g_n_*) >, where *l*_*x*_ ∈ *D*, *g*_*x*_ ∈ *G*. Radius *r* of every hyper-sphere *h* is denoted by *r*_*h*_ and *r*_*d*_ is a default radius value. Value of *r*_*h*_ is regenerated after every generation of size *S*.

**Output**: *R* is a triple < (*c*, *d*_*h*_, *g*_*h*_) >, here *c* shows center of hyper-sphere, *d*_*h*_ shows the density of hyper-sphere, and *g*_*h*_ ∈ *G* shows group of hyper-sphere. *n* denotes size of data-set *D*

1: Initialise *H* ← ∅, *d*_*h*_ = 0

2:  **for** *x* = 1, 2, …‥, *n* **do**

3:  *h*_*in*_ ← *false*

4:   **for** (*h* ∈ *H*_*g*_) **do**

5:    **if** (*d*(*l*_*x*_, *h*) ≤ *r*_*g*_) **then**

6:     *h*_*in*_ ← *true*

7:     Add point *l*_*x*_ in hyper-sphere *h* by updating center *c*_*h*_ and Density of hyper-sphere *d*_*h*_

8:     of hyper-sphere.

9:    **end if**

10:   **end for**

11:  **if** *h*_*in*_ ← *false* **then**

12:   **if** *x* == 1 **then**

13:    Create a new hyper-sphere *h* and compute center *c*_*h*_ of hyper-sphere from *l*_*X*_

14:    *H*_*g*_ ← *H*_*g*_ ∪ *h*

15:    Initialize *d*_*h*_ ← 1, *r*_*h*_ = *r*_*d*_

16:   **end if**

17:   **else**

18:    Create a new hyper-sphere *h* and compute center *c*_*h*_ of hyper-sphere from *l*_*X*_

19:    *H*_*g*_ ← *H*_*g*_ ∪ *h*

20:    Initialize *d*_*h*_ ← 1, *r*_*h*_ = *Sum*(*r*_*h*_)/|*H*_*g*_|

21:   **end if**

22:   **if** *x* == *S* **then**

23:   Update radius *r*_*h*_ of all hyper-spheres as the mean of existing radius and

24:   similarity between center of hyper-sphere and all the points that were added

25:   in that hyper-sphere during this generation.

26:   **end if**

27:  **end for**

A detailed interpretation of training algorithm 1 is given below:

- When this algorithm starts running it will initialize an empty set *H* containing all the hyper-spheres that will be created in training process. It will also set the density of hyper-spheres as zero as shown in line 1 of the algorithm.
- From line 2 to 27, the proposed algorithm runs a loop over all the data points in the data-set to put them into hyper-spheres. At line 3 it sets *h*_*in*_ variable to false to make sure each point will become part of at least one hyper-sphere.
- From line 4 to 10 a loop iterates over all hyper-spheres and calculates the distance of every data-point from the center of the hyper-sphere. At line 5 it checks if distance is less than or equal to the radius of the hyper-sphere then at line 6 it sets *h*_*in*_ as true showing that this point now resides in some hyper-sphere. Line 7 and 8 put that data point into a corresponding hyper-sphere and updates the center and density of the hyper-sphere.
- In line 11 the algorithm checks the value of *h*_*in*_. If it is false, there can be two possibilities either this is first point so *H* = *ϕ* or this point does not reside in any of the existing hyper-spheres. In the case of the first point the algorithm will create a new hyper-sphere and makes that point as the center of the hyper-sphere. It will also assign 1 as the value of the density of hyper-sphere and the radius will be set as the default radius value as shown at line 13 to 15. If a point does not reside in the existing hyper-spheres then a new hyper-sphere will be initiated but the radius is set as the average of the radii of all existing hyper-spheres as shown on line 20.
- The algorithm updates the radius of all updated hyper-spheres after a certain number of data-points called generation. At line 22, this algorithm checks the number of data-points processed so far and if it is equal to the size of generation then the algorithm will update the radius of all updated hyper-spheres as the average of their radius and the average distance of all the data-points that entered into a hyper-sphere during generation.

**Algorithm 2**: **NoR-MOOCs Recommendation**

**Input**: testing set < *l*_1_, *l*_2_ …, *l_n_* > consists of testing points. R is a triple < (*c*, *d*_*h*_, *g*_*h*_) > in which *c* is center, *d*_*h*_ is weight o, and *g*_*h*_ ∈ *G* is group of hyper-spheres. *r*_*g*_∀_*g*∈*G*_ shows dynamic radius of each group *g* ∈ *G*.

**Output**: *g* ∈ *G*

1: Initialise *h*_*g*_ ← ∅ ∀*g* ∈ *G*

2: *h*_*in*_ ← false, *s* ← ∅

3:  **for** < (*c*, *d*_*h*_, *g*_*h*_) > ∈ *R* **do**

4:   **if** (*d*(*l*_*n*_, *c*) ≤ *r*_*g*_) **then**

5:    *h*_*in*_ ← true

6:    *h*_*g*_ ← *h*_*g*_ ∪ *h*

7:   **end if**

8:  **end for**

9:  **if** *h*_*in*_ = *false* **then**

10:   **for** (*h* ∈ *H*) **do**

11:    Find distance of all hyper-spheres from the point *l*_*n*_.

12:    select hyper-sphere with least distance from test point and point it in *h*_*g*_.

13:   **end for**

14:   **for** (*h* ∈ *h*_*g*_)**do**

15:    Normalize distance by dividing it to to that density of that hyper-sphere.

16:    Compute recommendation by giving high weight to the hyper-sphere having high

17:    normalized distance value.

18:   **end for**

19:  **end if**

A detailed description of the recommendation algorithm 2 is given below:

- First the recommendation algorithm will initialize an empty set *h*_*g*_ that will contain all of the hyper-spheres in which a test point resides as shown on line 1.
- To keep track of whether a point matches any of the existing hyper-spheres or not a variable *h*_*in*_ is set to false as shown on line 2.
- From line 3 to 8 a loop is created over all existing hyper-spheres. In this loop the distance of the test point is measured with the center of all hyper-spheres and if the distance is less than the radius of the hyper-sphere then *h*_*in*_ is set to true and the hyper-sphere is added in *h*_*g*_ and now these hyper-spheres will be used for recommendation.
- At line 9 the algorithm checks the value of *h*_*in*_. If it is false that means this testing point does not exist in any of the existing hyper-spheres. In this case, it would select a hyper-sphere that is nearest from the test point and put it in *h*_*g*_ as shown on line 11 and 12.
- From line 14 to 18 a loop iterates over all the hyper-spheres in set *h*_*g*_. At line 15 it normalizes the distance with the density of a hyper-sphere then computes a recommendation by giving higher weighting to the hyper-sphere having a high normalized value as shown on line 16 and 17. To understand this, assume *P* shows the set of hyper-spheres in which *l*_*x*_ resides. The algorithm will take the density of hyper-spheres in the set *P* and normalize the value of the distance of that point by dividing it by the density. This is shown in the Eq 2.
  $$\begin{array}{c} Nor=\frac{d(c_{h},l_{n})}{f}, \end{array}$$ (2)
  The algorithm then sorts the values of *Nor* in descending order and computes results for the test point giving high weighting to the hyper-spheres having high *Nor* values while computing recommendations.

**Fig 10:**
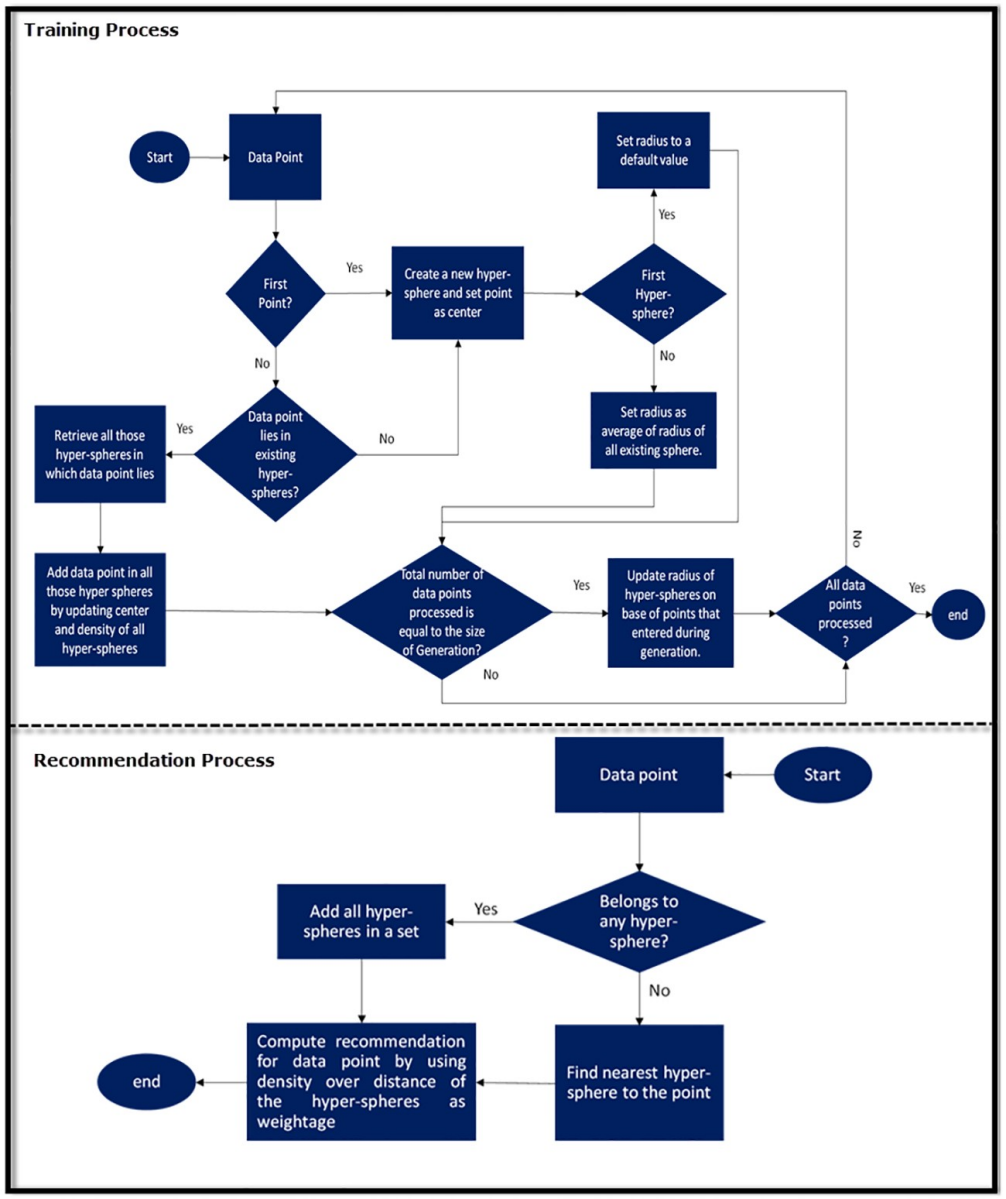
Flow diagram of training and recommendation process.

NoR-MOOCs normalizes the distance of every point with the density of that hyper-sphere because in this way it will obtain higher values for the hyper-spheres that are nearest to the test point and have high density. The rationale behind this is that a denser and nearer hyper-sphere can be a good source of recommendation for the test point. Fig 10 shows a flow diagram of the training and recommendation process. As shown in Fig 10, each data point is processed only once in the training process. While computing a recommendation the distance of every test point is normalized with the density of that hyper-sphere.

## 4 Experimental evaluation and results

### 4.1 Experimental setup

#### 4.1.1 Data set

To evaluate the working of the NOR-MOOCs, we used the COCO [34] data-set. This is a research-purpose-only data set, which includes data from Udemy https://www.udemy.com/., one of the global marketplaces for MOOCs. We used this data set because according to the best of our knowledge only this MOOC data set contains the rating of courses along with other attributes of courses [20].

In the COCO data set, each course can have no or many ratings while every learner should have rated at least one course. Table 1 shows the characteristics of the real COCO data set.

**Table 1:** Characteristics of COCO data-set.

Characteristics	COCO Dataset
Number of learners	2,546,865
Number of courses	43,113
Number of ratings	4,584,313
Rating scale	0 to 5
Sparsity	0.99583
Highest number of ratings given by a user	1159
Highest number of ratings given to a course	57,346

**Fig 11:**
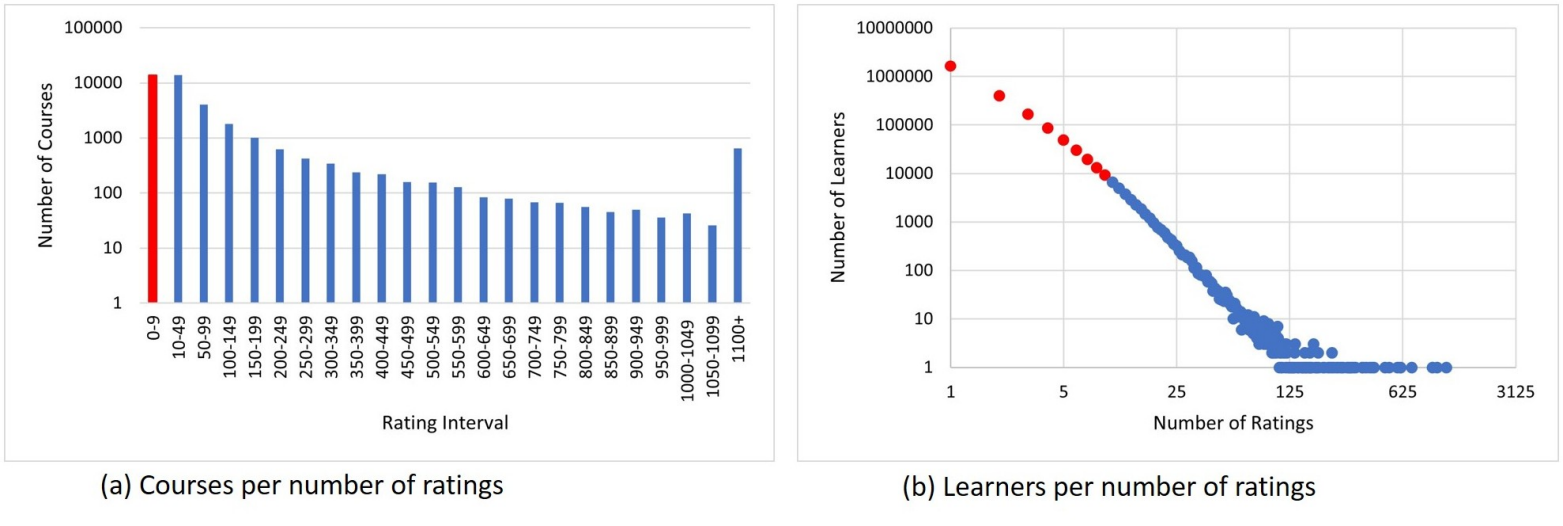
Rating distribution of COCO data-set. Highlighted data is removed to decrease sparsity.

To reduce the sparsity of the data set, we considered learners who have at least 10 ratings. After re-sampling the data set, there were 37*K* learners, 30*K* courses and 600*K* ratings. Fig 11a, shows the trend in the numbers of ratings per course. Although, the number of ratings per course is decreasing there are still a number of courses that have large numbers of ratings. Fig 11b shows the distribution of learners per the number of provided ratings. It can be observed from Fig 11b that a large number of learners have given less than 10 ratings. The red color in Fig 11 shows the data that we have removed to decrease sparsity.

#### 4.1.2 Evaluation methodology

For the evaluation of NOR-MOOC algorithm, we used 5-fold cross validation. We further divided the training set into training and validation sets to evaluate the sensitivity of the parameters. We used 80% of the data for training and 20% for validation.

#### 4.1.3 Performance evaluation metrics

In this study, we used predictive and classification accuracy metrics to benchmark our algorithm with traditional algorithms K Means and Collaborative Filtering based recommender systems [35]. Mathematical formulas of all the metrics are given in Table 2. A description of the metrics is as follow:

**Predictive accuracy metrics**: Predictive accuracy metrics measure how accurately the system predicts a course that a learner will rate, i.e. it shows how close the predicted rating is to the actual rating given by the learner. We have used Root Mean Square Error (RMSE) and Coverage to measure predictive accuracy of NoR-MOOCs algorithm.
  - Root Mean Square Error (RMSE) represents the square root of the differences between the predicted rating by NoR-MOOCs and the actual rating given by the user.
  - Coverage measures the proportion of courses from all courses in the data set that the system can recommend. It can be measured by simply calculating the percentage of all the courses that the system can recommend. It is a very important metric as a system having low coverage is less valuable because the system cannot give the recommendation for all the courses in the data set.
**Classification accuracy metrics**: Measures how many times a system accurately recommends a good item (the item that the user really likes) to the user. This evaluation metric is useful in the situation where the system has to recommend good items or lists of items to the user from the items available in the system. This metric includes Precision, Recall, Receiver Operating Characteristic (ROC) and F1 which we used for the evaluation of our algorithm.
  - Receiver Operating Characteristic (ROC) model tries to measure the level to which a recommender system can effectively differentiate between a good and a bad item. The ROC model considers only two classes for items i.e. good items (of interest to the user) and bad item (in which the user is not interested).
  - Precision is a measure of the total number of true positives divided by the total number of elements predicted.
  - Recall is defined as the number of true positives divided by the total number of samples.
  - F-measure (F1) is the harmonic mean of precision and recall. F-measure provides a value of 1 in the case of a perfect recommendation, whereas in the case of the worst recommendation, it gives a value of 0.

**Table 2:** Mathematical formulas of metrics that were used to evaluate the NoR-MOOCs algorithm.

Metric	Formula
RMSE	$\sqrt{\frac{1}{\left|D^{test}\right|}\sum_{i=1}^{\left|D^{test}\right|}{\left(p_{i}-a_{i}\right)}^{2}}$ *p*_*i*_ is the actual rating, *a*_*i*_ is the predicted rating and *D*^*test*^ is the test data.
Precision	$\frac{t_{p}}{t_{p}+f_{p}}$ here *t*_*p*_ is true positives and *f*_*p*_ is false positive.
Recall	$\frac{t_{p}}{t_{p}+f_{n}}$ here *t*_*p*_ is true positives and *f*_*n*_ is false negative.
F1	$\frac{2(PrecisionXRecall)}{Precision+Recall}$

We also designed a random recommender algorithm for the trained model and compared the remaining evaluation metrics with random recommender systems, K-Means and Collaborative Filtering.

### 4.2 Results

This section presents a detailed discussion on the experimental results.

#### 4.2.1 Tuning optimal parameters of rating based algorithm

Below we describe the tuning of important parameters i.e the similarity measure method, radius size and generation size.

**Fig 12:**
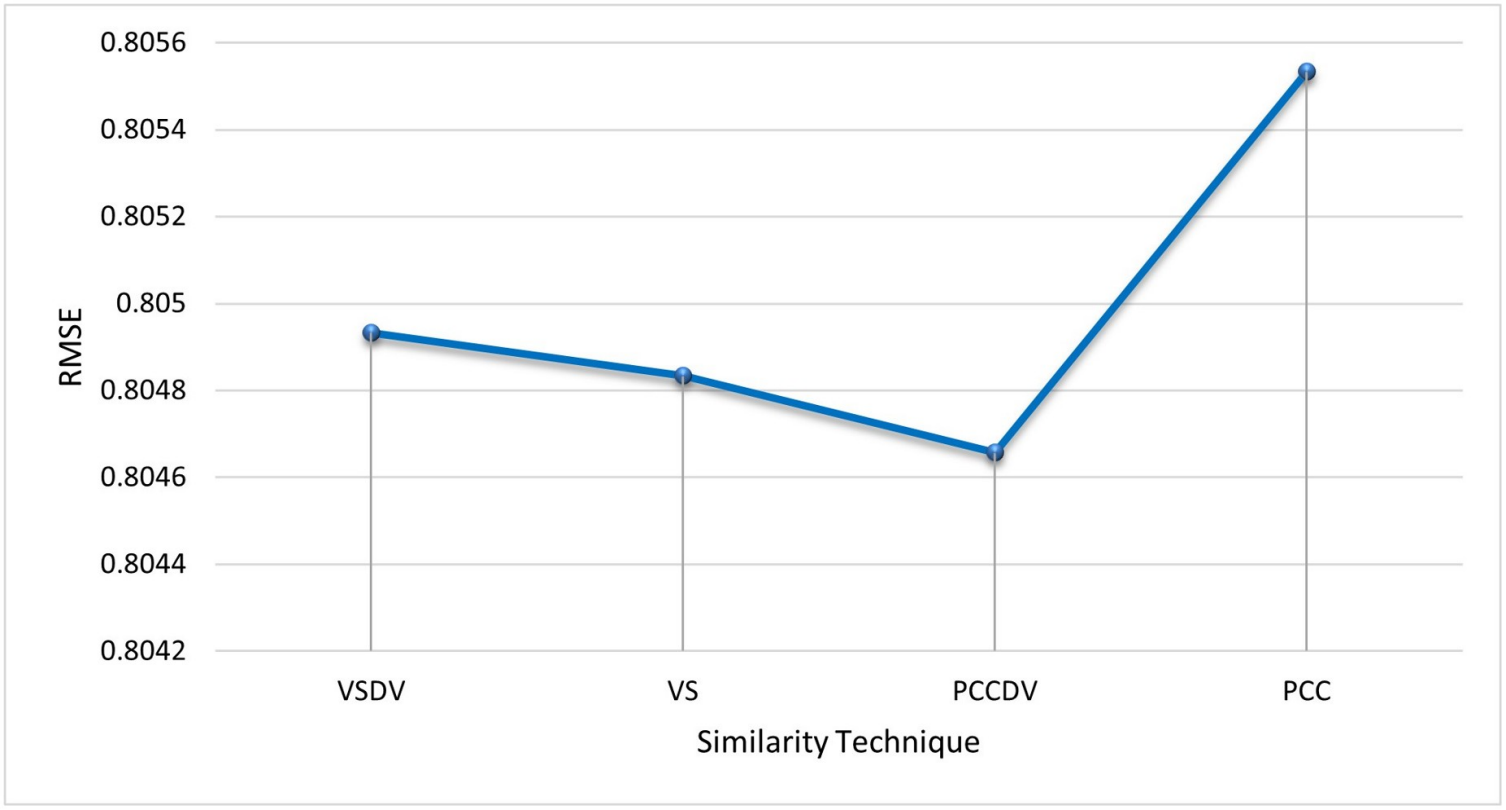
Comparison of PCC, PCCDV, VS and VSDV in term of RMSE. PCCDV gave best results.

**Fig 13:**
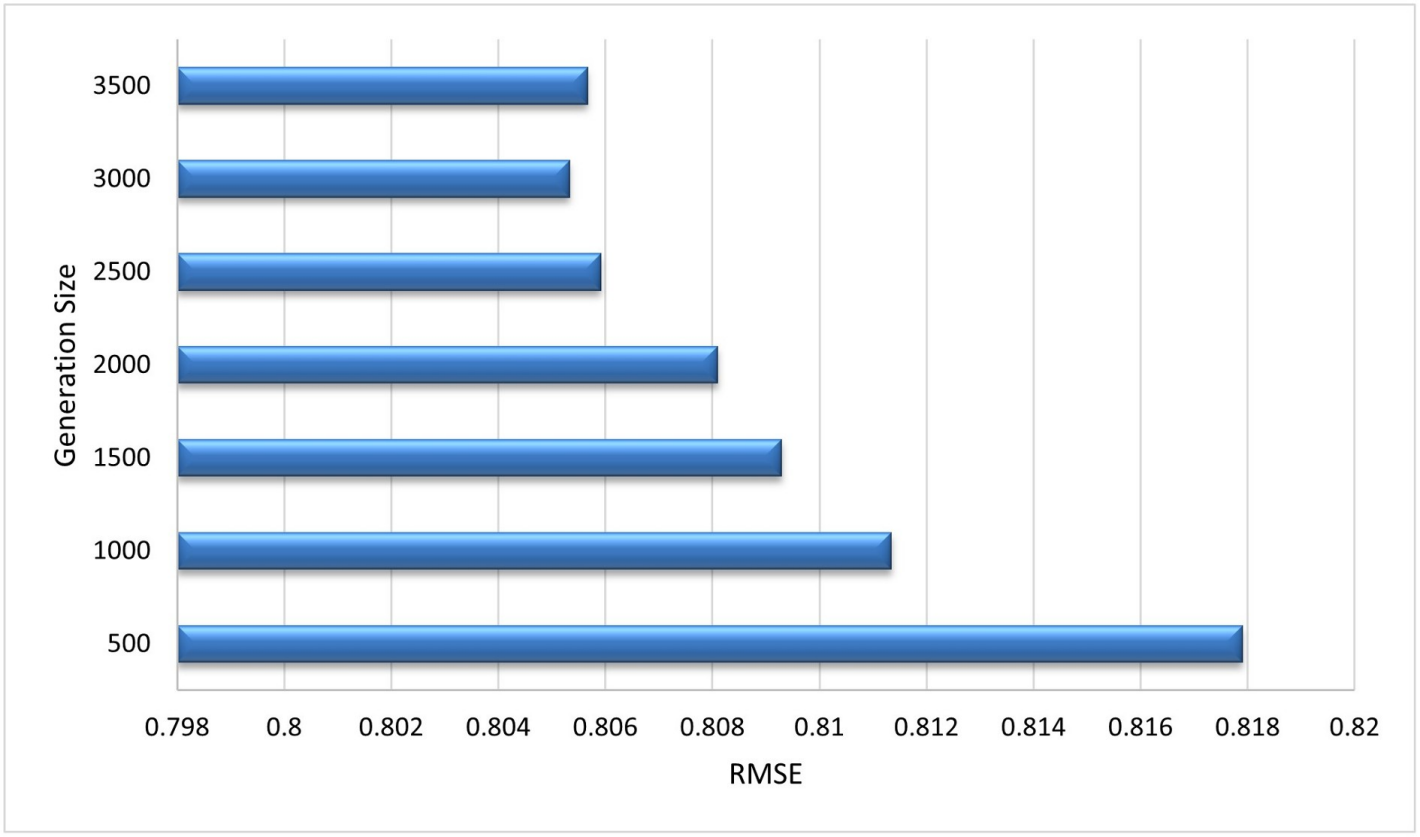
Tuning of generation size to get optimal results. A generation size of 3000 produced the best results.

**Fig 14:**
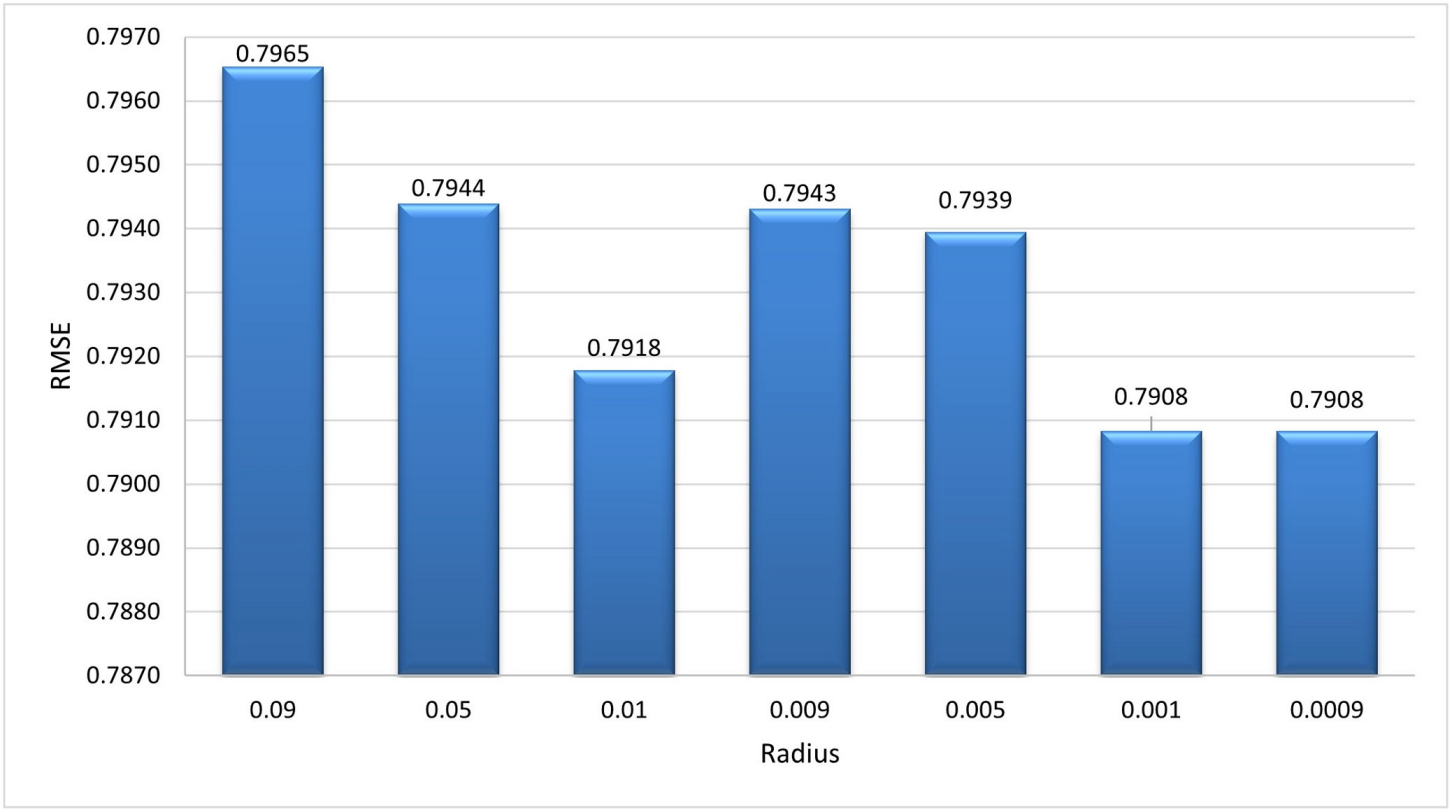
Tuning for the optimal radius value: It can be observed that at 0.001 our algorithm showed the best results.

- **Similarity Measure Methods** Different similarity measure methods are compared to choose the best results for the algorithm. The inverse of distance measure is used to compute similarity. Pearson’s Correlation distance measure (PCC), Pearson’s Correlation distance measure with default votes (PCCDV), Vector Similarity (VS), and Vector Similarity with default votes (VSDV) were compared [36]. Fig 12 shows the comparison of RMSE in all similarity techniques. PCCDV gave better results in terms of RMSE than the other techniques so this was used to compute similarity for the subsequent experiments.
- **Generation Size** After every generation, the radius of all hyper-spheres is recalculated on the basis of the number of points that enter the system during the generation. The size of generation is selected after tuning the algorithm to get optimal results. From Fig 13 it can be observed that the value of RMSE is very high for a small generation size, whereas with the increase in generation the value of RMSE started decreasing. Our algorithm showed best results at the generation size of 3000. We have used this size for remaining experiments.
- **Optimal Radius Value** When the first hyper-sphere is created, there is no way to calculate its radius so this algorithm uses a default value as the radius for the first hyper-sphere. Fig 14 shows tuning of the default value of the radius. RMSE is used to select the best value. It can be observed that value of RMSE is minimum at 0.001 and after that it became constant. So we select 0.001 as the optimal radius value.

#### 4.2.2 Performance comparison with K-Means and random recommender

We designed a random K-Means based recommender and a Collaborative Filtering based recommender system to compare results. We compared results in terms of Coverage, RSME, ROC, Precision, ReCall and F1 measure. Our algorithm out-performed K-Means and a random recommender in terms of Coverage, RMSE, and ROC as shown in Table 3. Coverage of the rating based algorithm is 60% greater than K-Means and Collaborative Filtering whereas it is 80% greater than a random algorithm. As the COCO dataset is very sparse, coverage and RMSE of NoR-MOOCs is good because it uses fuzzy clustering in that a data-point can belong to more than one hyper-sphere. This allows hyper-spheres to have a high number of data points which helps in generating accurate recommendations. On the other hand, K-Means keeps every data-point in one cluster which causes a decrease in the chance of getting a recommendation for every learner due to the sparsity of the data-set and it also decreases the quality of the recommendation. A Collaborative Filtering based recommender system utilizes ratings of similar users for computing recommendations which is greatly dependant on the sparsity of the data-set.

**Table 3:** Comparison of proposed algorithm with random, K-Means and Collaborative Filtering in terms of coverage, RMSE, and ROC.

Method	Coverage	RMSE	ROC
**NoR-MOOCs**	**99.99**	**0.7908**	**0.9367** ± **0.002639**
**K-Means**	39.37	0.8359	0.7478 ± 0.004676
**Collaborative Filtering**	33.40	0.8374	0.37254 ± 0.004554
**Random**	17.30	0.8123	0.7212 ± 0.005394

**Fig 15:**
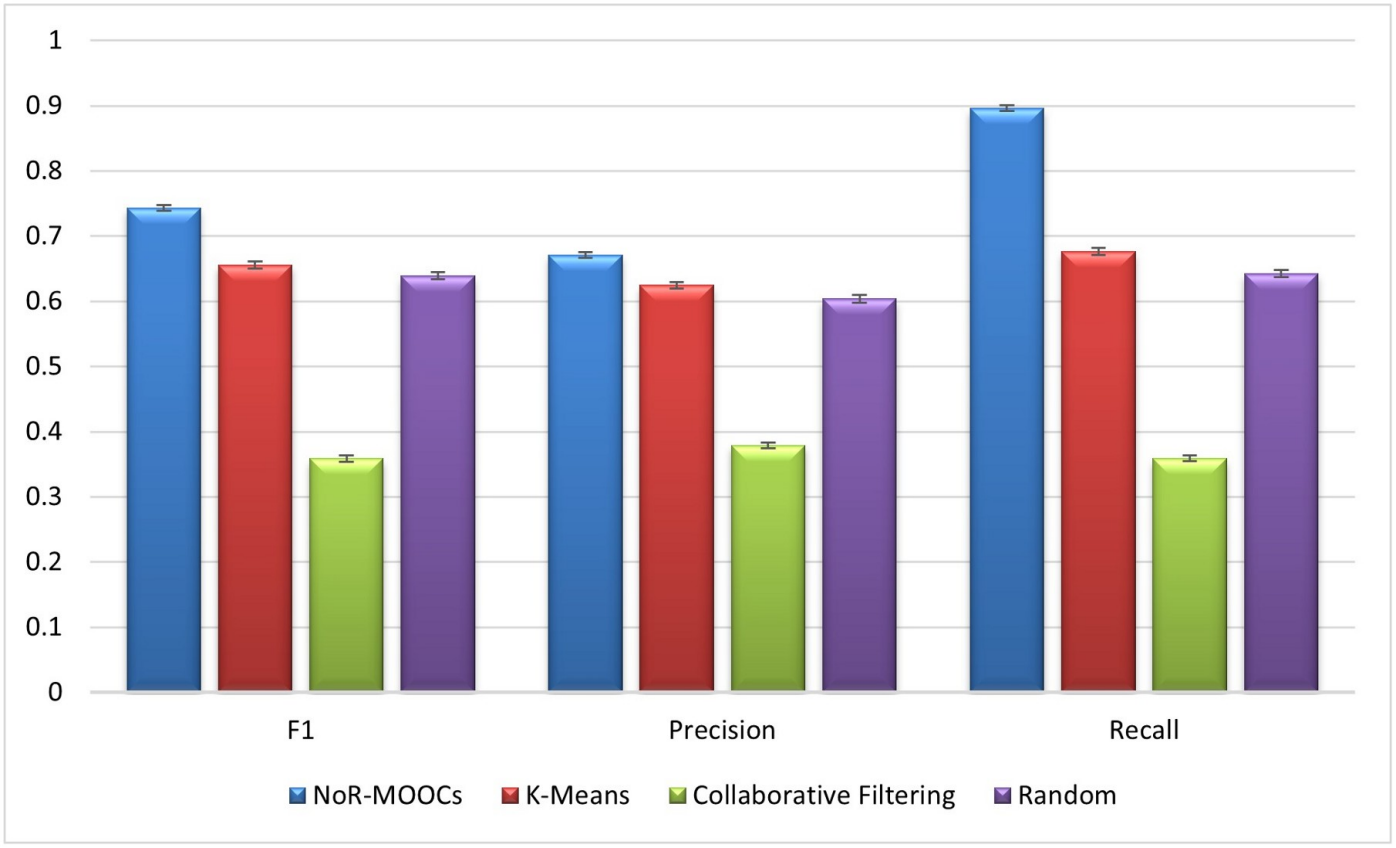
Comparison of F1, precision and recall of NoR-MOOCs, random, K-means algorithm and Collaborative Filtering.

RMSE and ROC of NoR-MOOCs are better than K-Mean because Nor-MOOCs gives weight to all related hyper-spheres and chooses the hyper-sphere with the higher number of data-points and the most similar hyper-sphere which helps in choosing the appropriate hyper-sphere for recommendation. Fig 15 shows precision, recall and F1 of all algorithms. The error bars in Fig 15 represent the standard deviations. From Fig 15 it can be observed that Recall of NoR-MOOCs algorithm is higher than that of the other three algorithms. Precision and F1 of NoR-MOOCs algorithm is also superior to the K-means, Collaborative Filtering and random recommender systems.

#### 4.2.3 Significance of the results

To evaluate the significance of the results, we performed a simple t-test and a paired t-test evaluation.

A t-test is a statistical test that is used to compare the means of two groups of values. It determines whether a hypothesis has an effect by comparing results of that hypothesis with actual values. In our case we have compared ratings recommended by our algorithm with actual ratings given by the user.

The two-tailed, paired t-test with a 99.9% confidence level has been applied to evaluate the performance of NoR-MOOCs, K-Means, Collaborative Filtering and a random algorithm. The results of the simple t-test and the paired t-test for all the algorithms are shown in Table 4. For *α* = ±0.001 and *df* = ∞ the results of the t-test and the paired t-test should be less then 3.090 and 3.291 respectively.

**Table 4:** Comparison of the simple t-test and paired t-test values of the proposed algorithm with k-means, Collaborative Filtering and random algorithm.

Test TypeMethod	NoR-MOOCs	K-Means	Collaborative Filtering	Random
**Simple t-test**	**2.95**	3.82	3.99	3.89
**Paired t-test**	**3.19**	4.93	5.22	5.11

Results of the proposed algorithms are within range of acceptable values to prove that the difference between the rating predicted by the algorithm and the actual rating is not significant.

#### 4.2.4 Complexity

The space complexity of our algorithm is also linear with the number of observations (e.g. course, learner and rating). We can decrease space complexity by using a sparse structure for storing the dataset. The time complexity of NoR-MOOCs is linear with respect to the number of clusters and the points in the cluster. The time complexity of the proposed algorithm is *O*(*nm*), where *n* < |*U*| is the average number of ratings given to a course and *m* < |*I*| is the average number of ratings by a learner.

While K-Means complexity is linear it is also iterative. Moreover, seed selection impacts its complexity and it may get stuck in local optima with random seeds, for more information please refer to our previous work [9].

In addition to time complexity, in certain cases, when there is large amount of data, K-Means requires extensive memory which might not be pragmatic.

## 5 Conclusion

Traditional recommendation algorithms have been used to make recommendations for learners in MOOCs. However, these systems do not scale well with the increase in data and moreover on the arrival of new data these systems need to be retrained making them costly in terms of time and storage. Therefore, these systems are not practical for recommender systems where data is increasing continuously, i.e. MOOC recommender systems. In this paper, a new algorithm has been proposed, namely a ‘rating based MOOC recommender system’, that is incremental and scales well with available data. These experimental results show that the NoR-MOOCs algorithm is more accurate, scalable, and has better coverage compared with traditional systems. Batch algorithms such as K-Means and Collaborative Filtering give good results but these algorithms are very expensive and require retraining with the arrival of new data points.

NoR-MOOCs is very flexible and can work with different types of data. The results of the rating based algorithm are better than K-Means and a random algorithm; however, the algorithm still requires further improvement. Different variants of this algorithm can be designed to further improve the results. Radius is one important parameter of this algorithm that could be calculated using different methods to improve the results. One potential avenue of future research is to improve this algorithm so that it can perform recommendations on the basis of learners’ characteristics (e.g. demographics and behaviour).
